# Supplementary figures and images for: Slowing deforestation in Indonesia follows declining oil palm expansion and lower oil prices
Source: PLoS One. 2022 Mar 29;17(3):e0266178. doi: 10.1371/journal.pone.0266178 (PMC8963565; doi:10.1371/journal.pone.0266178)

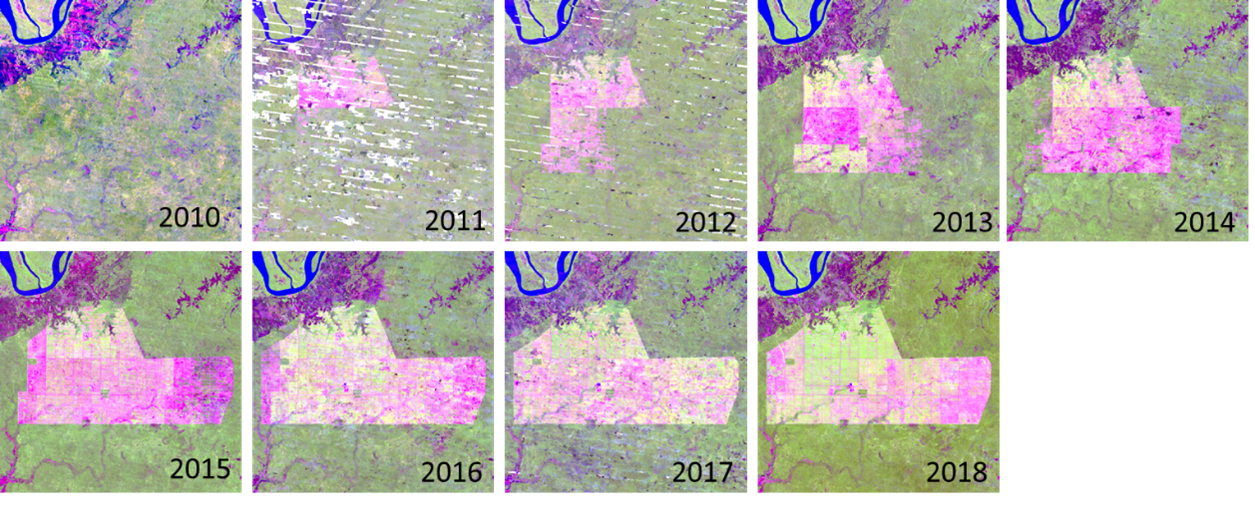

Supplement: S1 Fig — These images reveal the annual expansion of an industrial plantation. Imagery displayed in false colors (RGB: Short-wave infrared: band 5; Near infrared: band 6; Red: band 4). Here, forest appears green, while recently cleared areas appear pink. (TIF) [file pone.0266178.s001.tif]

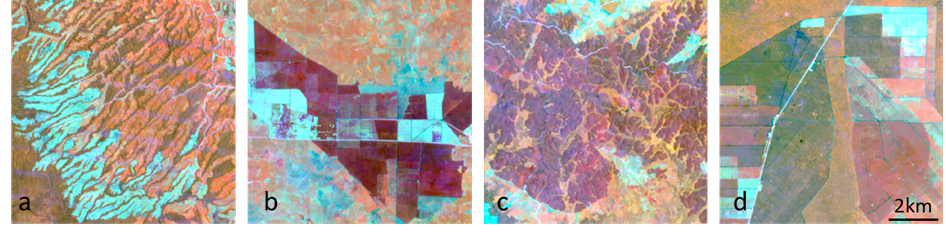

Supplement: S2 Fig — Imagery displayed in false colors (RGB: Near infrared; Short-wave infrared; Red). Closed-canopy acacia stands appear red to dark red. Recently harvested stands appear bright cyan. forest is dark brown. (a) Network of riparian forest in an acacia plantation on steep terrain. (b) a plantation on flat surface with rectilinear network of roads and canals. (c) a plantation on steep terrain with fewer forest corridors. (d) a plantation of flat peat swamps, with acacia stands of varying age, and rectilinear network of canals and roads. (TIF) [file pone.0266178.s002.tif]

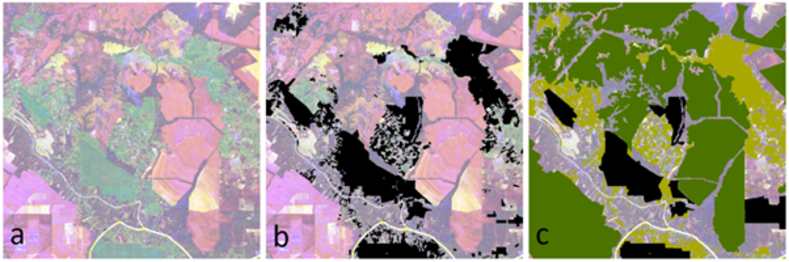

Supplement: S3 Fig — Imagery displayed in false color composite (RGB: VV,VH, Red). (a) Closed-canopy (mature) oil palm plantations appear green because of the higher backscatter than other vegetation types in the dual cross-polarization bands (VH). (b) Auriga’s oil palm base map (black) missed several plantations (green). (c) The final map used the radar data to capture missed oil palm plantations: industrial oil palm (black); smallholder oil palm (light green); industrial acacia plantations (dark green). (TIF) [file pone.0266178.s003.tif]

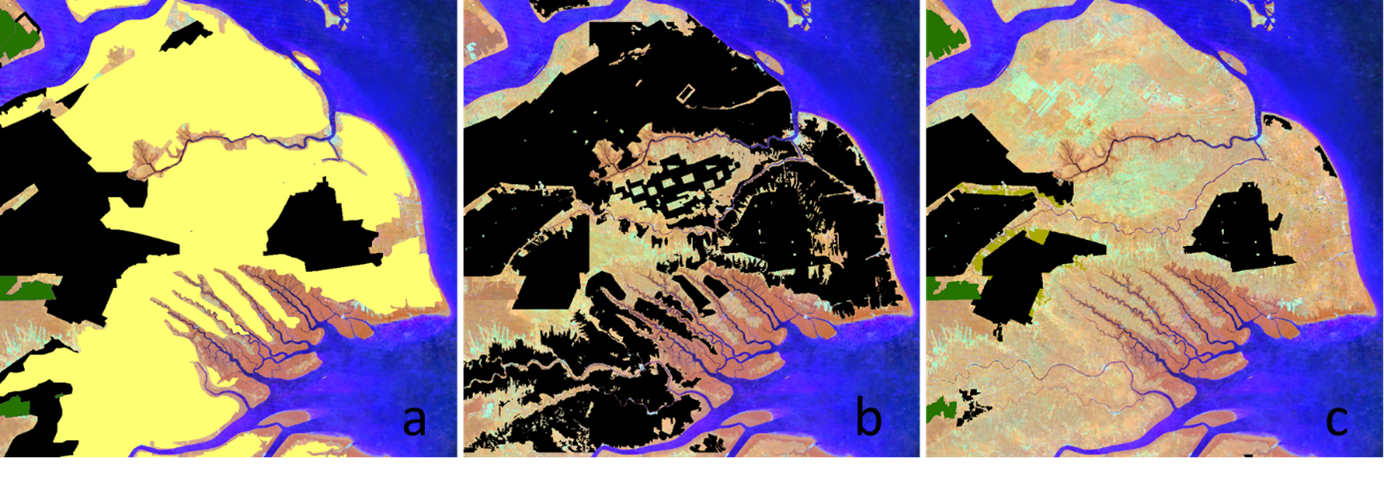

Supplement: S4 Fig — (a) provincial government map of oil palm (black) coconut (yellow), and acacia (green). (b) Auriga oil palm base map. (c) In the final map the areas misclassified as oil palm have been removed. (TIF) [file pone.0266178.s004.tif]

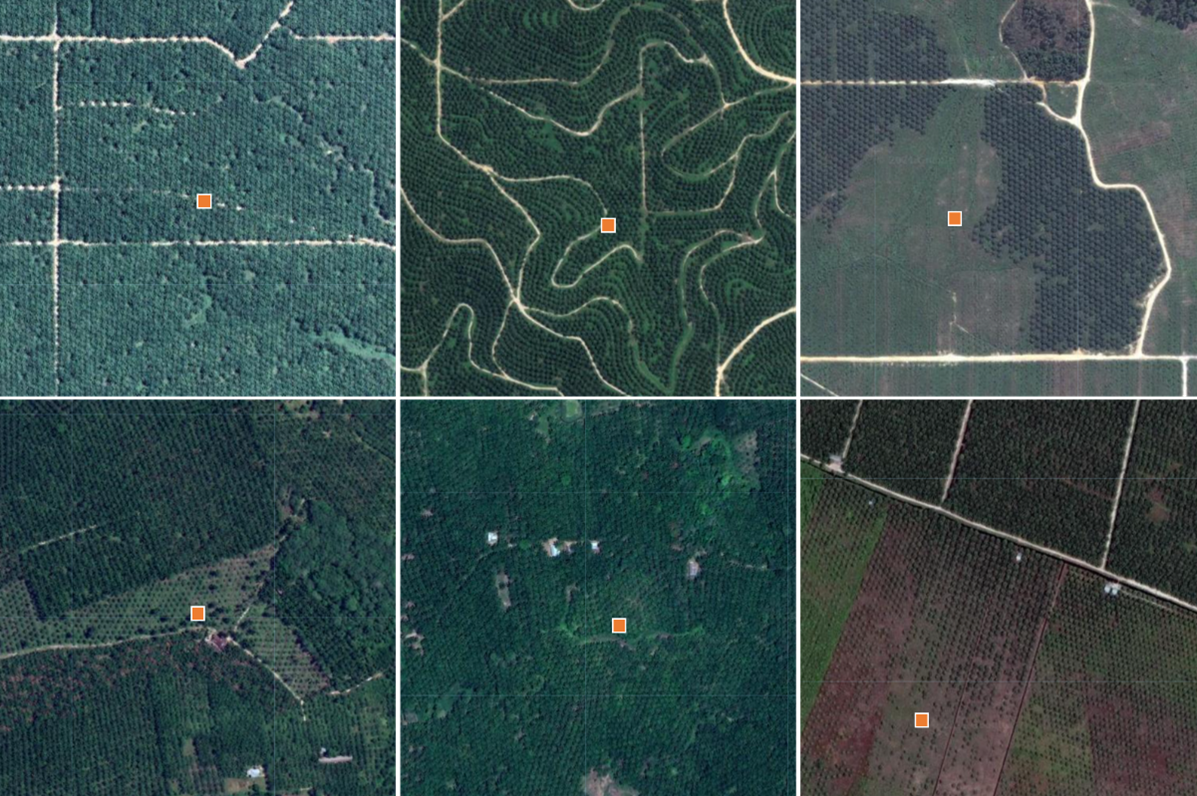

Supplement: S5 Fig — The top images show the distinct planting patterns of three types of industrial plantations: mature (closed-canopy) plantation on flat surfaces, with rectilinear trails (left), mature plantation on undulating surfaces, with contour trails (middle), and partly damaged plantation (right). The bottom images show the distinct planting patterns of smallholder plantations: young (open-canopy) plantation on flat surface (left), mature (closed-canopy) plantation (middle), and damaged plantation on flat surface (right). (TIF) [file pone.0266178.s005.tif]

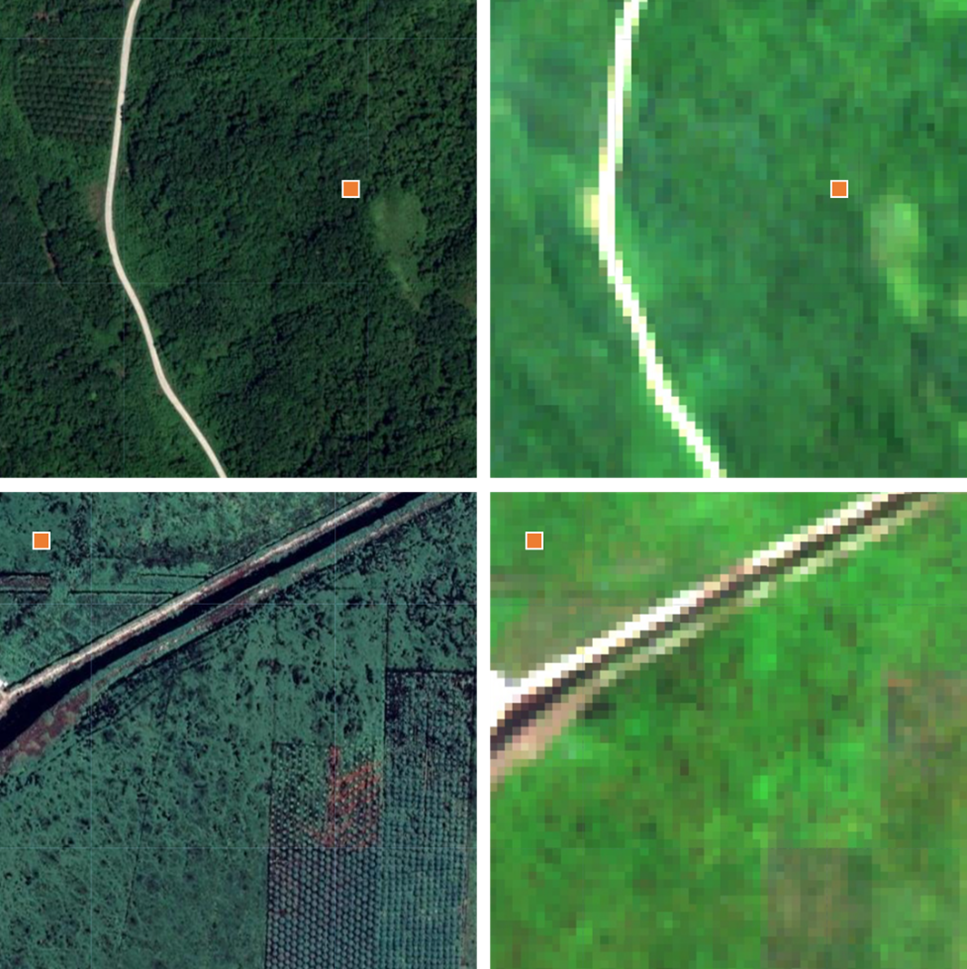

Supplement: S6 Fig — We observed no change in land cover between high-resolution imagery (<1 m) taken before 2019 (left images) and 2019 Sentinel-2 composite (10 m) (right images). The sites were labelled ‘other’. (TIF) [file pone.0266178.s006.tif]

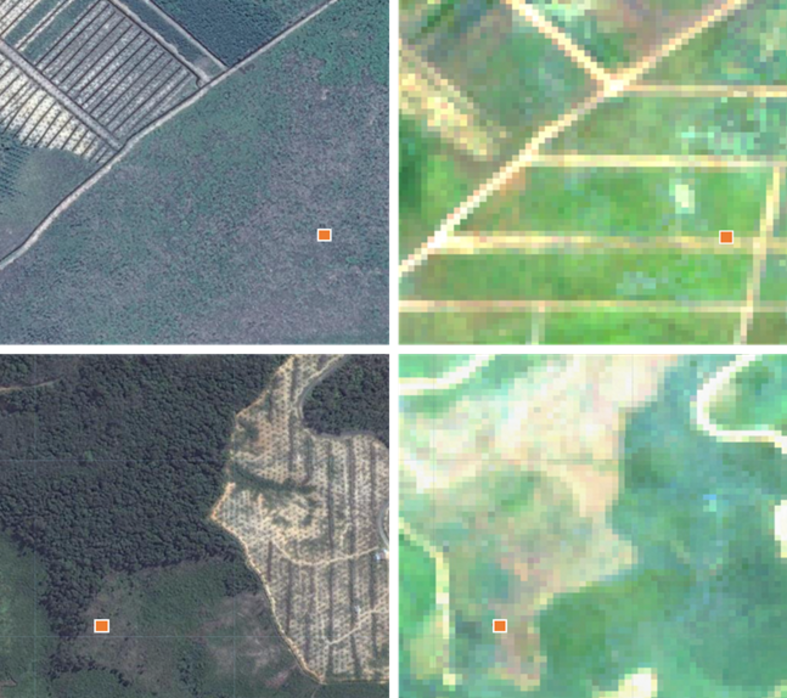

Supplement: S7 Fig — We observed change in land cover between high-resolution imagery (<1 m) taken before 2019 (left images) and 2019 Sentinel-2 composite (10 m) (right images). In the top images, clearing typical of industrial plantations (rectilinear grids) appears in 2019. We labelled this site ‘industrial oil palm’. In the bottom images, clearing typical of smallholder plantations appears in 2019, and is adjacent to an existing smallholder oil palm plantations seen on the high-resolution imagery. We labelled the site ‘smallholder oil palm’. (TIF) [file pone.0266178.s007.tif]

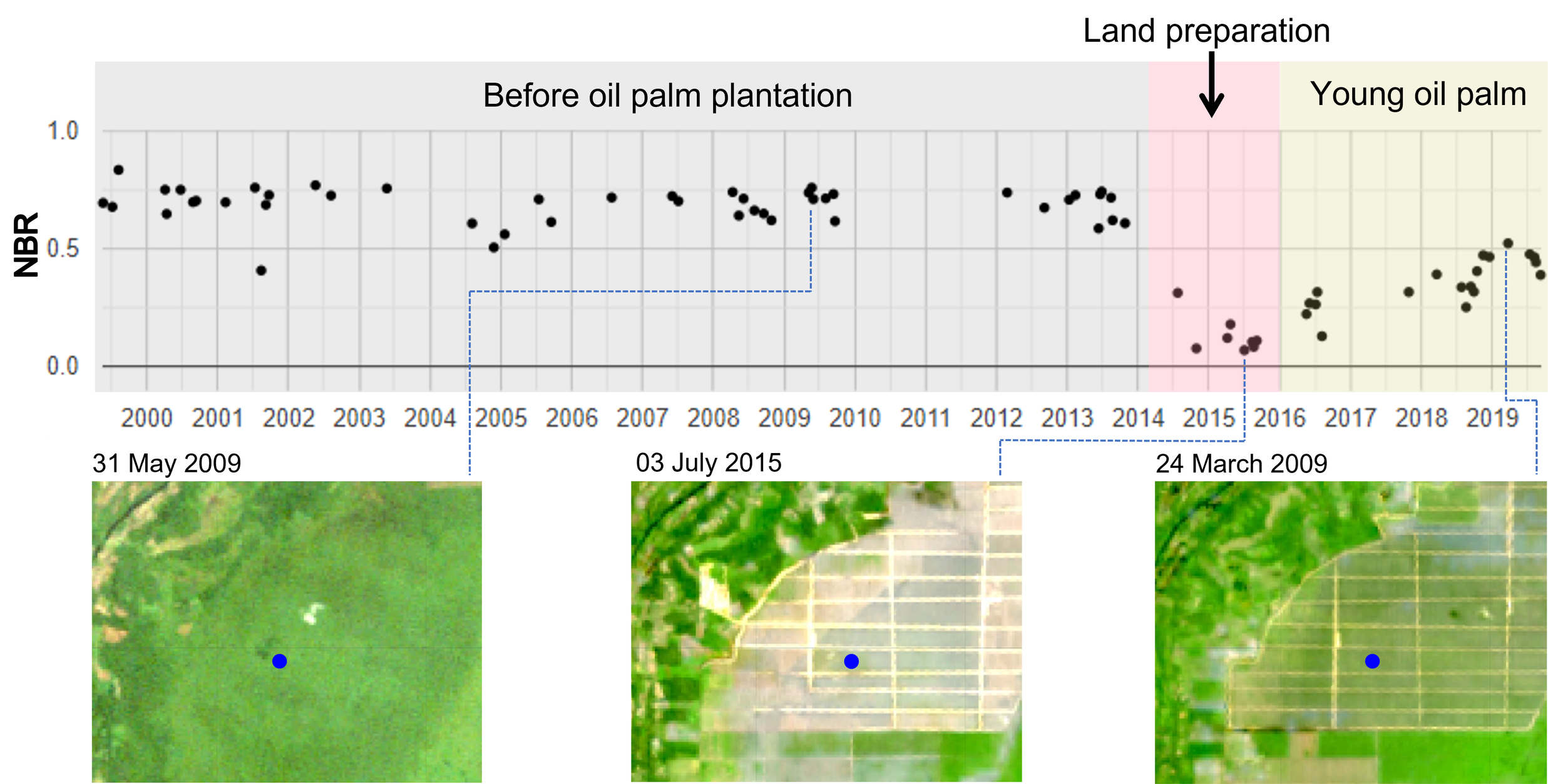

Supplement: S8 Fig — The visual interpretation of original time-series Landsat images corroborated that the area was converted to industrial oil palm plantation in 2014. (TIF) [file pone.0266178.s008.tif]

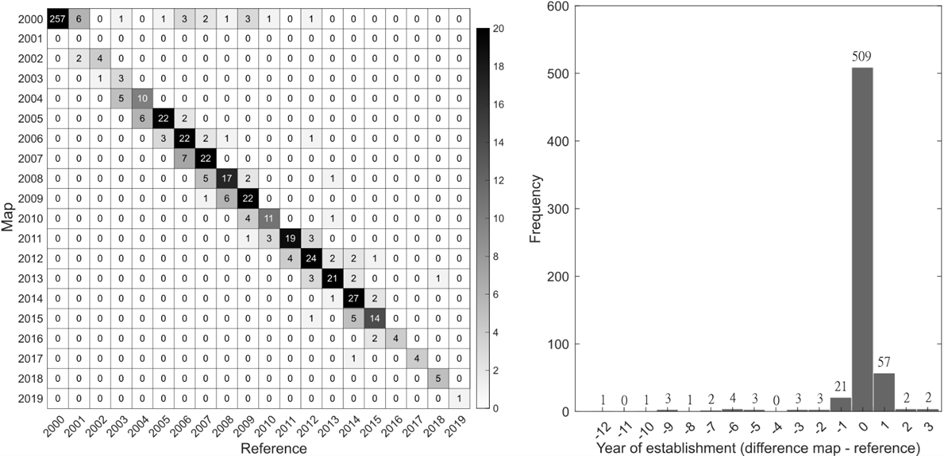

Supplement: S9 Fig — Revealing the correspondence between the year of establishment of industrial oil palm plantations verified with Landsat images and the year of establishment reported on the map for all reference sites labelled ‘Industrial oil palm’ (N = 612). (TIF) [file pone.0266178.s009.tif]

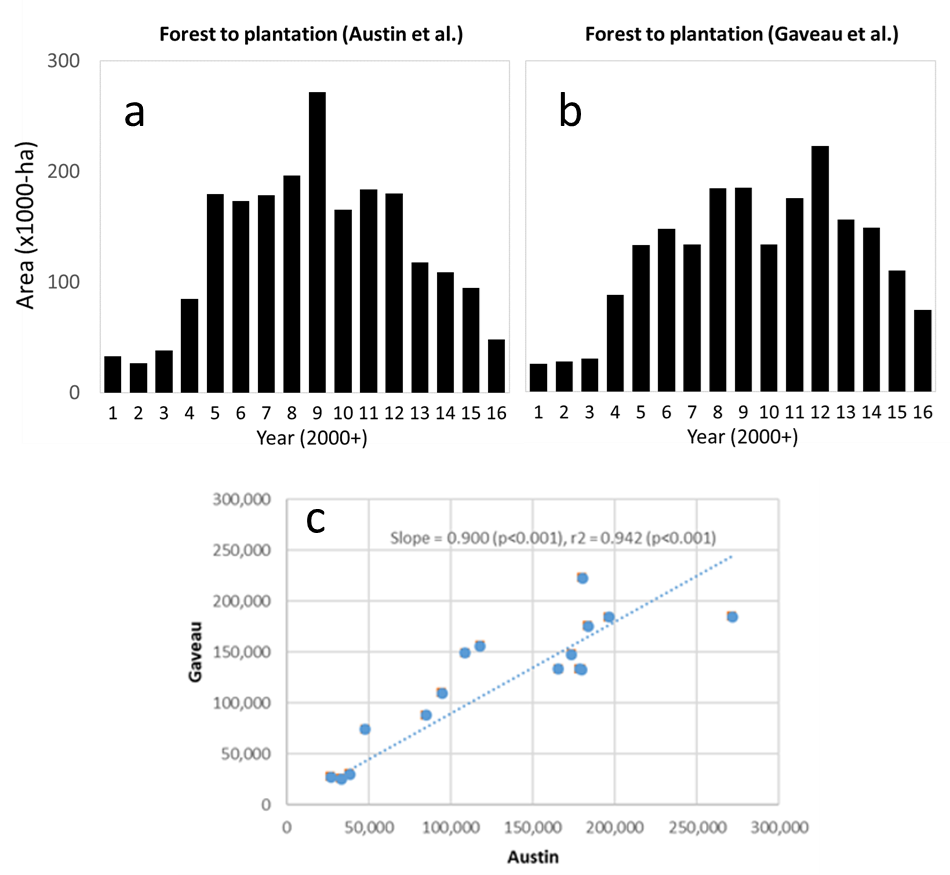

Supplement: S10 Fig — (a) Based on samples (Austin et al. 2019 [10]). (b) Based on wall-to-wall mapping (this study). (c) Shows the correspondence between both studies. The line represents the fitted regression model that goes through the origin (zero intercept). (PNG) [file pone.0266178.s010.png]

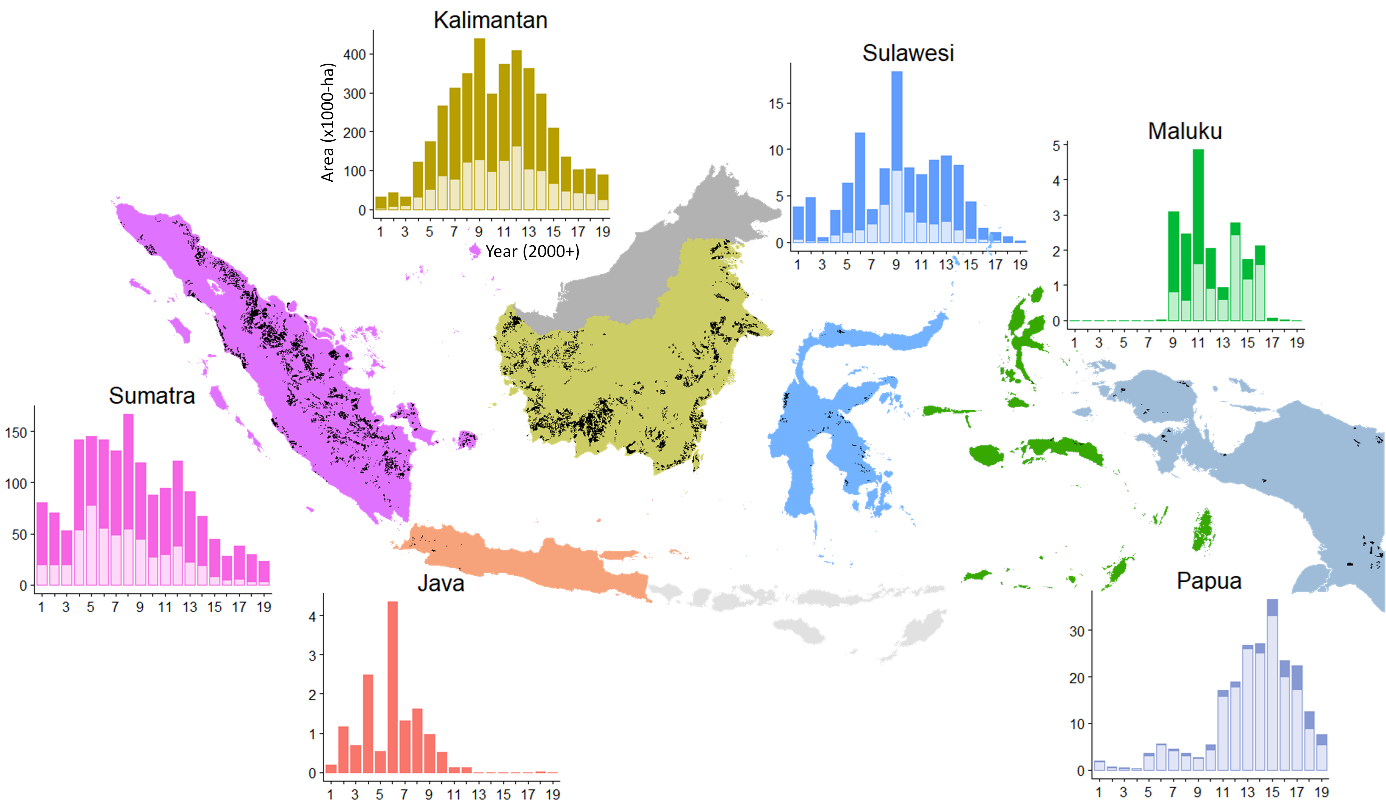

Supplement: S11 Fig — Y-axis represent areas (in 1000-ha, note different scales) of the total area of plantations added each year between by directly clearing forests (light bars, below), or by using areas already cleared (dark bars). Black areas on the map represent the total area of industrial oil palm plantations in 2019. (PNG) [file pone.0266178.s011.png]

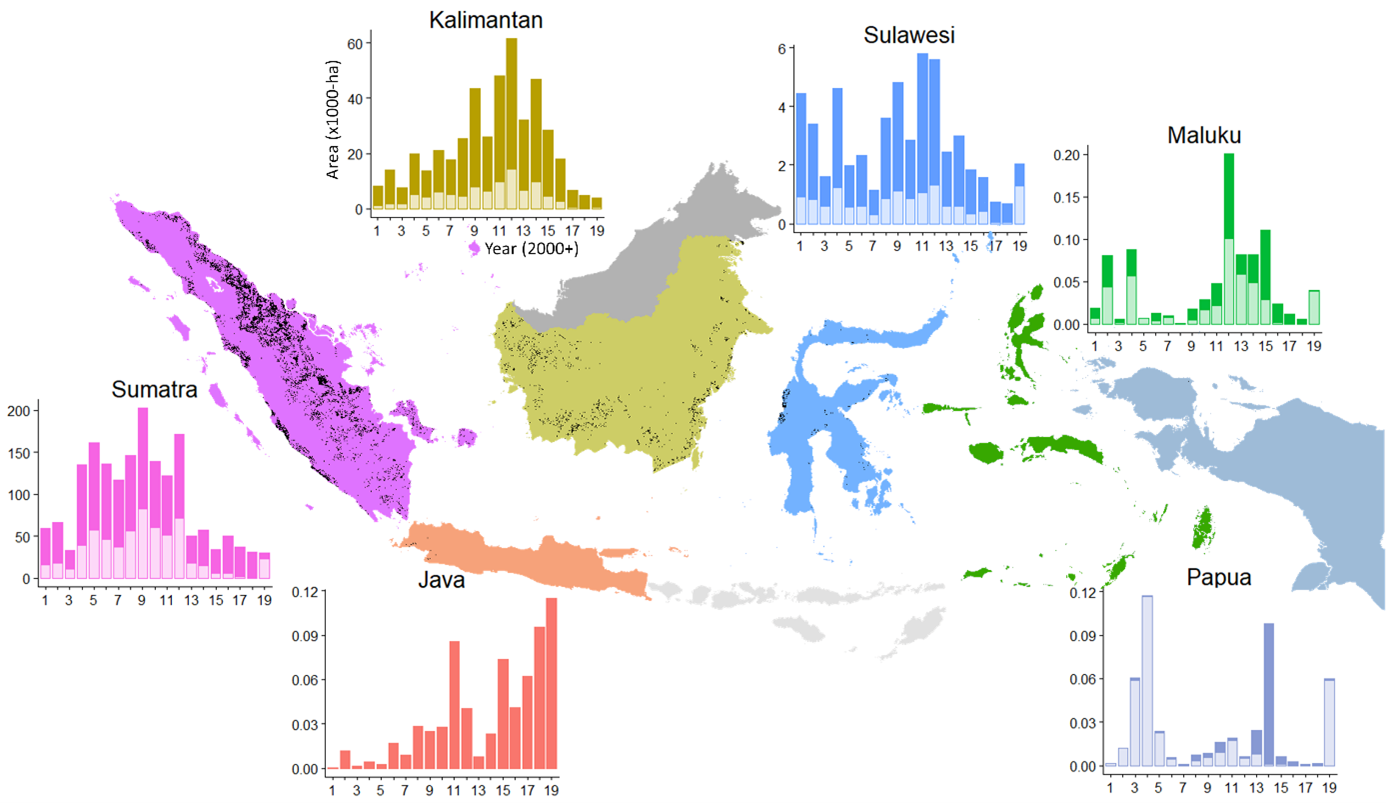

Supplement: S12 Fig — Y-axis represent areas (in 1000-ha, note different scales) of the total area of plantations added each year between by directly clearing forests (light bars), or by using areas already cleared (dark bars). Black areas on the map represent the total area of smallholder oil palm plantations in 2019. (PNG) [file pone.0266178.s012.png]
