# Supplementary material for: Slowing deforestation in Indonesia follows declining oil palm expansion and lower oil prices
Source: PLoS One. 2022 Mar 29;17(3):e0266178. doi: 10.1371/journal.pone.0266178 (PMC8963565; doi:10.1371/journal.pone.0266178)
Supplement: S1 Table — Description of sample data as an error matrix of reference sites counts (see S2 Table for recommended estimated error matrix used to report accuracy results). (DOCX) [file pone.0266178.s013.docx]

**S1 Table. Error Matrix.** Description of sample data as an error matrix of reference sites counts (see Table S2 for recommended estimated error matrix used to report accuracy results).

|  |  |  | | *Reference* | | | |  |
| --- | --- | --- | --- | --- | --- | --- | --- | --- |
|  |  | *Other* | *Industrial* | | *Smallholder* | *Total* | *A_m_ [ha]* | *W_h_* |
| *Map* | *Other* | 2314 | 21 | | 72 | 2407 | 90,302,754 | 0.848 |
|  | *Industrial* | 16 | 605 | | 14 | 635 | 10,316,986 | 0.097 |
|  | *Smallholder* | 43 | 7 | | 348 | 398 | 5,920,061 | 0.056 |
|  | *Total* | 2373 | 633 | | 434 | 3440 | 106,539,801 | 1 |
