# Supplementary material for: Slowing deforestation in Indonesia follows declining oil palm expansion and lower oil prices
Source: PLoS One. 2022 Mar 29;17(3):e0266178. doi: 10.1371/journal.pone.0266178 (PMC8963565; doi:10.1371/journal.pone.0266178)
Supplement: S2 Table — Description of sample data as an error matrix of reference sites populated by estimated proportions of area. (DOCX) [file pone.0266178.s014.docx]

**S2 Table.** **Error matrix.** Description of sample data as an error matrix of reference sites populated by estimated proportions of area.

|  |  | *Reference* | | | | |  |  |  |
| --- | --- | --- | --- | --- | --- | --- | --- | --- | --- |
|  |  | *Other* | *Industrial* | *Smallholder* | *Total* | *A_m_ [ha]* | | | *W_h_* |
| *Map* | *Other* | 0.815 | 0 | 0.025 | 0.848 | 90,302,754 | | | 0.848 |
|  | *Industrial* | 0 | 0.092 | 0.002 | 0.097 | 10,316,986 | | | 0.097 |
|  | *Smallholder* | 0.006 | 0.001 | 0.049 | 0.056 | 5,920,061 | | | 0.056 |
|  | *Total* | 0.823 | 0.101 | 0.076 | 1 | 106,539,801 | | | 1 |
