# Supplementary material for: Slowing deforestation in Indonesia follows declining oil palm expansion and lower oil prices
Source: PLoS One. 2022 Mar 29;17(3):e0266178. doi: 10.1371/journal.pone.0266178 (PMC8963565; doi:10.1371/journal.pone.0266178)
Supplement: S3 Table — (DOCX) [file pone.0266178.s015.docx]

**S3 Table.** **Share of deforestation caused by oil palm and pulp&paper expansion from 2001 to 2019 for Indonesia and by region.**

| Area (in Ha) | Indonesia | Sumatra | Kalimantan | Papua | Sulawesi | Java Maluku |
| --- | --- | --- | --- | --- | --- | --- |
| Landmass | 189,130,128 | 47,467,842 | 53,498,290 | 41,227,232 | 18,627,593 | 21,135,660 |
| Forest area 2019 | 87,758,114 | 12,063,230 | 25,742,162 | 34,289,462 | 9,114,005 | 5,871,624 |
| Forest loss 2001-2019 | 9,789,448 | 4,075,312 | 4,023,971 | 748,640 | 715,737 | 213,487 |
| Forest converted to pulp&paper and oil palm | 4,093,479  (42%) | 2,007,419  (49%) | 1,817,807  (45%) | 208,842  (28%) | 46,782  (7%) | 12,629  (6%) |
| Forest converted to oil Palm | 3,094,882  (32%) | 1,242,345  (31%) | 1,593,260 (40%) | 200,161  (27%) | 46,782  (7%) | 12,629  (6%) |
| Rapid conversion to oil palm | 2,849,796  (29%) | 1,166,806 (29%) | 1,434,493 (36%) | 194,996 (26%) | 43,319  (6%) | 10,181  (5%) |
| Rapid conversion to industrial oil palm | 2,129,301  (22%) | 553,480  (14%) | 1,341,610 (33%) | 194,671 (26%) | 29,807  (4%) | 9,733  (4.5%) |
| Rapid conversion to smallholder oil palm | 720,495  (7%) | 613,326  (15%) | 92,884  (2%) | 325  (0.0004%) | 13,512  (2%) | 448  (0.002%) |
| Rapid conversion to pulp&paper | 959,774  (10%) | 742,911  (18%) | 208,331  (5%) | 8,532  (1.14%) | 0 | 0 |
| 2019 oil palm  *Indus/small (%)* | 16,237,047  *64/36* | 9,486,516  *49/51* | 6,044,517  *84/16* | 272,808  *99/1* | 374,686  *55/45* | 58,520  *83/17* |
| 2019 pulp&paper | 2,966,181 | 2,016,570 | 939,874 | 9,737 | 0 | 0 |

We used a sinusoidal projection to calculate areas.

(%) of forest loss.

Pulp&paper stands for Acacia and Eucalyptus plantations
