# Supplementary material for: Slowing deforestation in Indonesia follows declining oil palm expansion and lower oil prices
Source: PLoS One. 2022 Mar 29;17(3):e0266178. doi: 10.1371/journal.pone.0266178 (PMC8963565; doi:10.1371/journal.pone.0266178)
Supplement: Attachment — Submitted filename: Reply to reviewers of PlosOne_17FEB2022.docx [file pone.0266178.s016.docx]

We thank the reviewers for their constructive comments. In most cases we have addressed their concerns as suggested. In a few cases we have acknowledged the issue and sought an alternative clarification or to make our intentions clearer. There are few examples where there may be a misunderstanding, but in each case we have reviewed/revised to make our text and views simpler to follow. In each case we have replied point by point. In finalising the manuscript, we have also reviewed it several times for coherence and content and thus made minor changes that benefit the text. We believe the revised article is substantially improved. We have included page numbers and line numbers in the revised manuscript. Below, we answer clarify against each point from the reviewers.

Reviewer #1: General comments:

I initially declined to review this paper, citing my lack of expertise in land use change detection and quantification, as well as having no background in economics. I did however state I was willing to review as a general non-subject-expert tropical forest ecologist, and I accepted the second review invitation under that condition. I advise the Subject Editor to make sure the other reviews are from subject-matter experts, and to consider my review as addressing more basic aspects common to any scientific paper.

As a general scientific reader (see caveats above) I found the paper interesting and readable. It addresses an important topic and is in general well-written and clearly presented.

We are grateful for the positive assessment.

Data are stated to be available “for interactive viewing” at a website. However, it would be impossible for a reader to know exactly what data from that website were used, and there is no guarantee that those data will be available over the long term. I suggest that authors provide the exact data that were used in their analyses, either in a separate data deposition on Dryad or the like, or as Supplemental Information. Websites and their databases change and disappear, whereas depositing the exact data used to produce this paper on Dryad or as SI “freezes” the exact data used in this paper. Just providing a web link for interactive viewing definitely does not comply PLOS One’s data availability policy of providing the “data required to replicate all study findings reported in the article, as well as related metadata and methods”.

We posted the oil palm expansion dataset on the data repository Zenodo at: <https://doi.org/10.5281/zenodo.6069212>

This manuscript was difficult to review because it did not follow PLOS One formatting guidelines, which require line numbers: “Include page numbers and line numbers in the manuscript file.” Lacking line numbers to refer to, I used several words of the text in brackets instead; the authors will have to use text search to find the section I’m referring to. References in the text also do not follow PLOS One format (brackets instead of superscripts).

We apologize for this oversight. We have included page numbers and line numbers in the revised manuscript and references in the text now follow Plos One format.

Specific comments:

The title seems logically reversed. The assertion is that lower palm oil prices drive slower deforestation and oil palm plantation expansion, so the more logical would seem to be
“Lower palm oil prices are associated with decreased deforestation and oil palm expansion in Indonesia”

We thought about this and discussed it with all the authors. Given the logic of the study itself, focusing on forest loss as our subject, and inferring the financial pressures that cause it, we prefer it the way it is. If we accepted the suggestion, we feel we would need to say much more about palm oil prices up front as that becomes the subject.

The distinction between industrial and smallholder farms should be quantified when it is Introduced in the Introduction. What is the upper bound in ha for small holder, and the lower bound in ha for an industrial plantation?

We have better defined industrial and smallholder plantations in the Introduction, and now provide an indication of their size in the results section. We have also moved the figure that characterizes different types of plantations from supplementary files into the main text a Fig 1.

Putting Materials and Methods after Results is permitted by PLOS One manuscript guidelines. My question to the Authors though is, does that make sense? How could one intelligently read a Results section without first reading the Methods? If one has to read the Methods to understand the Results, why not put the Methods first so the reader can read the paper in logical order?

We moved the (revised) Methods section before Results as suggested.

[Smallholder plantations are smaller] – Give some indication of the size range, for example a mean + SEM in ha.

We now provide an indication of their size in the results section. “The total area developed as industrial plantations reached 10.32 Mha (64%) in 2019 (minimum size of a plantation = 84 ha; max = 185,000 ha; mean = 3,000 ha). The total area developed as smallholder plantations reached 5.92 Mha (36%) in 2019 (minimum size of a plantation = 0.29 ha; max = 149,000 ha; mean = 49 ha)”. We note that the maximum size for smallholders corresponds to a large homogenous landscape made up of several small plantations.

Table 1. Define “industrial” in “rapid conversion to industrial”

We rephrased: Rapid conversion to industrial OP. and added a footnote under Table 1 to describe OP: Oil Palm

Reviewer #2: Oil palm expansion in the Southeast Asia plays an important role in forest conversion in the region. Spatiotemporal analyses of changes in oil palm distribution and deforestation help quantify this role and contribute to scientifically sound management of the land. There are existing studies and mappings of oil palm expansion in Indonesia, and this manuscript tries to improve such studies by providing time-series mapping of oil palm distribution and separating industrial and smallholder oil palm plantations. The results highlighted declining oil palm expansion and slowing deforestation, which are in general aligned with the existing studies.

1) Time-series mapping of smallholder oil palm plantations is an important contribution of the manuscript; however, the approach is questionable. The authors used an existing map of smallholder oil palm plantations in 2016-2018 and the time series of tree loss maps to derive the annual distributions back to the period of 2001–2019. This method implicitly assumes that all the distributions in the years of 2001 – 2019 are bounded by the existing map in 2016 – 2018. This assumption needs to be justified.

Yes that is correct. In the now revised Methods, we explain that, although the smallholder oil palm map (derived from the work of Indonesian NGO AURIGA) represented the situation in 2016-2018, we updated it to 2019 using radar data. We note, however that this 2019 update may be incomplete because radar imagery does not detect young plantations (< 3 years) well. We thus verified and quantified this underestimation through a rigorous validation. We added three Asterisks (*) above the values of the smallholder expansion for 2017, 2018, and 2019 in Fig 2c where Asteriks (*) indicate that the area of smallholder expansion is likely underestimated (see Figure legend; Fig 2). In discussion, we concluded that given this uncertainty, we cannot be certain that our study would detect every slight fluctuation in planting rates, such as for example a small increase in smallholder oil palm planting after 2016.

In the revised Methods, we explain the process in more detail: that using an existing map of smallholder oil palm plantations in 2019 (not 2016-2018 since we updated it to 2019, but this update is imperfect, see above) and the time series of tree loss maps to derive the annual distributions back to the period of 2001–2019 assumes that all the spatial distributions of expansion in the years of 2001 – 2019 are bounded by the existing 2019 smallholder map. The assumption seems reasonable given the nature of the crop.

2) The delineation and classification of two types of oil palm plantations are mostly relying on the visual interpretation of satellite images. In such case, survey to local experts is suggested to verify the mapping results.

We have explored different ways to cross check these data. We believe the most reliable experts are the ones who have done this assessment making it unclear what other “local experts” would offer (if there were discrepancies the source of the difference would be ambiguous). The authors of this study have published extensively on oil palm and possess extensive knowledge in the field of oil palm mapping both from the ground and from remote sensing. The interpreters working for the NGO AURIGA were all local experts and carried out several drone surveys at key smallholder landscapes. The study also includes concession data. Having said that we have explained our approach clearly.

3) Current ground truthing is mostly based on the checking of 3440 pixels in 2019. Since the manuscript focuses on the time series mapping in 2001 – 2019, the ground truthing pixels/year should be spread to the different time periods such as the beginning, the middle, and the late periods of 2001 – 2019.

We have made substantial revisions to ensure a robust validation of our time-series of industrial plantations. We added an analysis to verify the year the plantations were established. We visually interpretated Landsat time-series images acquired between January 2000 and December 2019 over the reference sites labelled ‘Industrial oil palm’ (N=612) to verify the year industrial plantations were established. The visually interpreted Landsat images were obtained from the Landsat-5, Landsat-7, and Landst-8 surface reflectance datasets. For each reference site, we plotted the values of the normalized burned ratio (NBR), an index ranging from -1 to +1, and looked for a sudden drop in the NBR time series, indicating a disturbance in the vegetation, possibly a conversion to oil palm (S8 Fig). We reviewed the images before and after the drop in NBR to verify whether the disturbance was a clearing event to establish oil palm. If the border of the cleared area was linear or rectangular, and grid or contour-like trails appeared on the sequence of Landsat images, this indicated an area converted to an industrial oil palm plantation, and we recorded the year of conversion (S8 Fig). Finally, we analyzed the correspondence between the year of conversion observed by visual interpretation and the year of conversion of our oil palm map.

We stress the excellent validation results for industrial oil palm extent in 2019 and expansion (2001-2019) and demonstrated the replicability of our time series against another study: a near one-to-one correspondence of our industrial time series against the Austin et al. dataset from 2001 to 2016. We demonstrate an underestimation for smallholder oil palm in 2019. We acknowledge that uncertainties remain for the validation of the smallholder plantations expansion. This is inescapable given data limitations. Landsat time-series imagery cannot determine the year smallholder plantations were established reliably because clearing small clearing patterns resemble other smallholder activities. We explored available time-series high resolution imagery on Google Earth for the 433 smallholder reference points scattered across the palm producing regions. However, these images are patchy and are not available every year. For example, if we found that an image taken in 2014 showed oil palm trees, we still could not know say when the plantation was established if earlier images were not available. This analysis was therefore inconclusive. We have done what is possible given data limitations, but we are confident that the overall trends and patterns are meaningful. In our revision we ensure these uncertainties and imperfections are clearly acknowledged and described.

We deposited our full oil palm expansion dataset at: <https://doi.org/10.5281/zenodo.6069212> to permit verification and reproducibility of our results.

4) The Kappa statistic is a more reliable index on classification accuracy, which is missing in the results.

We have included the kappa statistics. In Results, we report: “The overall accuracy of our 2019 mapped plantation extent is 95.6% (CI: 95.3%-96.0%) and Cohen’s Kappa coefficient is 0.85.”

Reviewer #3: This is a superbly executed and clearly written and presented study on a very important topic. The manuscript provides long-overdue clarity on patterns of forest loss and palm oil expansion, including for smallholder palm oil, for the last 20 years. I have only a couple of minor comments for revision. There are obvious challenges in mapping such a complex land use system, especially for smallholders, but the authors are to be commended on employing such a comprehensive approach to validation and clearly presenting both commission and omission errors.

We are grateful for the positive assessment and the recognition that we have achieved useful results in a challenging context.


Two small comments

1. You report on Page 13 that “We were more concerned to note that Xu et al. (2020)14 reported a peak in expansion in 2016 but subsequent discussions with those authors suggested an artefact due to using multiple data sources with distinct and sometimes inconsistent properties (Xu et al. pers. comm. to Gaveau 2020).” Given the importance of this contrast and unpublished nature of the reference it would be helpful to include more detail on where the “sometimes inconsistent properties” are evident in the analysis by Xu et al.?

Yes. We have reworded this part. In the data repository (Zenodo) where they published their dataset, Xu et al. have added a note recommending users to exclude the last year map (2016) in further analysis. Instead of citing our personal communication with Xu et al, we cite the dataset, where this recommendation is made:

Xu Y, Yu L, Ciais P, Cheng Y, Gong P. Annual oil palm plantation maps in Malaysia and Indonesia from 2001 to 2016. In: Zenodo, editor. 2019. <https://doi.org/10.5281/zenodo.3467071>

*Note:
The post-processing for the initial mapping results would cause the overestimation of changes in the last year (2016 in this case) map, therefore the authors recommend excluding the last year map in the further analysis.*

2. Page 17. Worth noting that as of 2021 the payment for results agreement between Norway and Indonesia has been terminated by Indonesia

Yes. We have added a comment.
